# A post-traumatic stress disorder among internally displaced people in sub-Saharan Africa: a systematic review

**DOI:** 10.3389/fpsyt.2023.1261230

**Published:** 2023-11-03

**Authors:** Tura Koshe, Mohammedamin Hajure Jarso, Mandaras Tariku Walde, Jemal Ebrahim, Aman Mamo, Adem Esmael, Lema Fikadu Wedajo, Solomon Seife, Mustefa Mohammedhussein, Desalegn Nigatu, Gebiso Roba Debele, Wubishet Gezmu

**Affiliations:** ^1^Department of Nursing, College of Health Sciences, Madda Walabu University, Shashemene, Ethiopia; ^2^Department of Psychiatry, College of Health Sciences, Madda Walabu University, Shashemene, Ethiopia; ^3^Department of Psychiatry, College of Health and Medical Sciences, Haramaya University, Harar, Ethiopia; ^4^Department of Psychiatry, College of Health Sciences, Madda Walabu University, Robe, Ethiopia; ^5^Department of Midwifery, College of Health Sciences, Mattu University, Mattu, Ethiopia; ^6^Department of Midwifery, College of Health Sciences, Madda Walabu University, Shashemene, Ethiopia; ^7^Department of Nursing, College of Health Sciences, Mattu University, Mattu, Ethiopia; ^8^Department of Public Health, College of Health Sciences, Mattu University, Mattu, Ethiopia

**Keywords:** post-traumatic stress disorder, internally displaced persons, systematic review, sub-Saharan Africa, 2023

## Abstract

**Introduction:**

Despite the prevalence of post-traumatic disorder in internally displaced persons, which is well established, and the fact that respective international organizations are working on the issues, little attention is given in the context of sub-Saharan Africa, This study aims to review the available data about the prevalence and determinants of post-traumatic stress disorders among internally displaced people in sub-Saharan Africa.

**Methods:**

Studies published in the English language that have a clear outcome of interest and are available in full text were included. Six electronic databases were searched to identify published studies on the prevalence and determinants of posttraumatic stress disorder among IDPs in sub-Saharan Africa. This includes PubMed/MEDLINE, Scopus, EMBASE, PsychInfo, and the Web of Science. All relevant studies till June, 2023 were assessed. The review was done as per the Preferred Reporting Items for Systematic Reviews and Meta-analyses (PRISMA-2009) and registered on PROSPERO (CRD420222997111).

**Results:**

Originally, 33,138 articles were found in six databases, and finally, eleven studies were reviewed. The prevalence of post-traumatic stress disorder in sub-Saharan African countries was disproportionately presented in this review, ranging from 12.3% in Central Sudan to 85.5% in Nigeria. From a total of 11 studies, eight of them reported more than 50% of the magnitude of post-traumatic stress disorder, pointing to a higher magnitude of the prevalence of post-traumatic stress disorders in the region. The study identified numerous factors that contributed to post-traumatic stress disorder among the internally displaced population. Female gender, depression, anxiety, stress, being single, low level of educational status, experiencing or witnessing traumatic events, and psychological trauma were evidenced for their association with post-traumatic stress disorder.

**Conclusion:**

These results demonstrate a higher prevalence of post-traumatic stress disorder compared to other regions of the world. The participants’ socio-demographic characteristics, including age, being single, being female, and a low level of education, were identified as factors contributing to PTSD. Moreover, the review identified that depression, anxiety, and experiencing or witnessing traumatic events were also influencing factors for PTSD among IDPs. The concerned bodies need to reinforce the monitoring and evaluation of the mental health programs of IDPs in the region.

**Systematic review registration:**

https://www.crd.york.ac.uk/prospero/display_record.php?RecordID=299711, CRD42022299711.

## Introduction

In places where conflict, war, violence, and natural disasters are frequently observed, displacement from these areas to the safe zone is expected (1). Worldwide, more than 40 million people have been displaced from their primary living areas (2). A person or group of people who have been forced or obliged to flee or leave their homes or places of habitual residence, in particular as a result of most of the internally displaced people being accounted for by human-made causes like wars, violence, and ethnic conflicts. The impacts of these events not only change socio-political structures but also have a lasting impact on people’s migration (3).

During the events, victimized internally displaced persons experienced a variety of mental health problems, particularly depression and post-traumatic disorders (4). Post-traumatic stress disorder is a mental disorder associated with witnessing and being exposed to stressful life events, including murder threats, kidnapping, loss, and starvation (4). More importantly, if the displacements are prolonged, the person will experience more behavioral problems (5). The results of a systematic review among internally displaced persons revealed that the pooled prevalence of post-traumatic disorder (PTSD) was (3–88%) (3) and (4.4–86%) (6).

Sub-Saharan Africa is one of the regions where human and natural disasters are prominently reported (5). Evidence reported indicates that the prevalence of post-traumatic disorders in sub-Saharan African countries among internally displaced people varies. Accordingly, 12.3% in Sudan (7), 54% in Uganda, and 80.2% in Kenya (8) 31.6% of the male population and 40.1% in the West Nile region (9), 24.8% in Rwanda, 78% (10), and 42.2% (11) in Nigeria, 58.4% (12), and 67.5% (13) in Ethiopia, and 32% in Mogadishu, Somalia (14).

Post-traumatic stress disorder is more common in married people, women, people who have been displaced more than once, people who have depressive symptoms, people who have witnessed the murder of family members and the destruction of property, people who are unemployed, and people who are in their forties (11). Despite the well-established prevalence of post-traumatic disorder in internally displaced people and the respective international organizations working on the issues, little attention is given to reducing conflict, civil war, violence, and other trauma in Africa. Therefore, this study is aimed at reviewing the available data about the prevalence and determinants of post-traumatic stress disorders among internally displaced people in sub-Saharan Africa.

## Methods and material

### Study protocol and registration

The International Prospective Register of Systematic Reviews (PROSPERO) has recorded the protocol for the systematic review and meta-analysis under the accession number CRD42022299711. The Preferred Reporting Items for Systematic Review and Meta-analysis (PRISMA) were used to construct the methodology for this systematic review and meta-analysis.

### Data source and search strategy

This study concludes previously reported studies on the prevalence of posttraumatic stress disorder among IDPs in sub-Saharan Africa. Accordingly, six electronic databases were searched to identify research studies published on the prevalence of posttraumatic stress disorder among IDPs in sub-Saharan Africa. This includes PubMed/MEDLINE, Scopus, EMBASE, PsychInfo, and the Web of Science. Google Scholar was used to search for the relevant gray literature. The details of the database search result are stated in Appendix 1. A combination of the medical subject headings and keywords was also used as the search terms. All relevant studies till June, 2023 were assessed.

**Figure 1:**
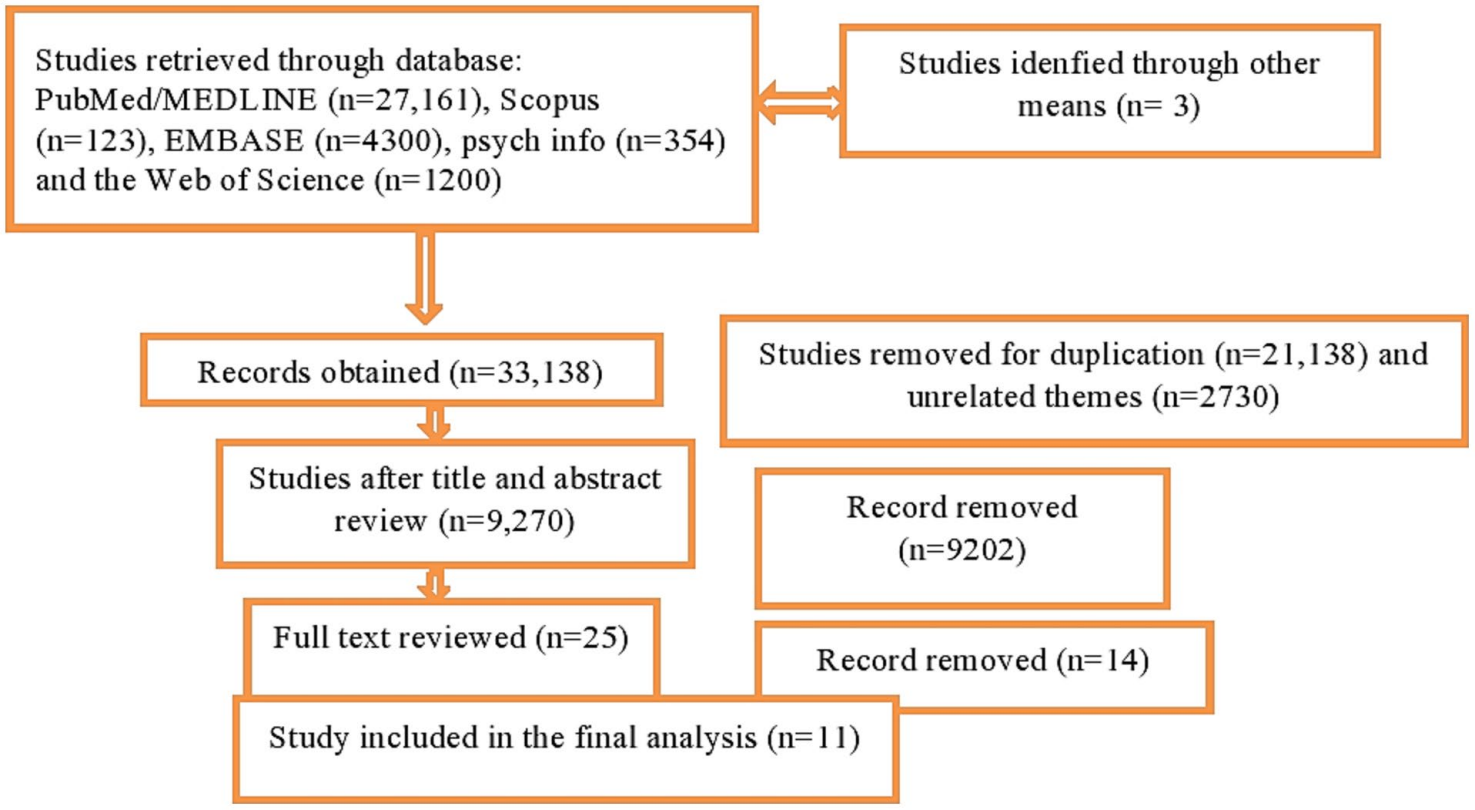
PRISMA flow chart displaying the selection process of identified studies.

The search terms used include (posttraumatic stress disorder) OR (posttraumatic stress symptoms) OR (post-traumatic stress disorder) OR (posttraumatic stress symptomatology) OR (PTSD) AND (internally displaced persons) OR (internally displaced peoples) OR (forced migration) OR (internally displaced survivors) OR (IDP) AND (Africa) OR (Sub-Saharan Africa) OR (sub-Saharan African countries) AND (#1 AND #2 AND #3). The reference lists of the included studies were manually searched for further relevant literature. The details of articles or studies obtained from different databases are stated in Figure 1.

### Eligibility criteria

The review was conducted according to the Population, Exposure, Comparison, Outcome, and Study Design (PECOS) guidelines. The Population (P) included in this review were all segments of the population (regardless of sex and age) living in sub-Saharan Africa. Being internally displaced was considered Exposure (E). The review compared (C) the prevalence and associated factors of PTSD among internally displaced people in sub-Saharan African countries with the prevalence elsewhere in the region. The outcome (O) of the current review was the prevalence of PTSD. Regarding the study designs, the study included all the quantitative observational studies (cross-sectional or survey, case–control, cohort or longitudinal studies, randomized controlled trials, and quasi-experimental designs). Studies published in the English language that have a clear outcome of interest and are available in full text were included. Published and unpublished studies, including case reports, commentaries, reviews, editorials, and conference abstracts, and a study with an unclear outcome or inadequate information were excluded.

### Screening and selection process

The selection process of the studies included in the current review was undertaken by two authors (MM and MH) and used an organized checklist called PICOS (participants, intervention/exposure, comparison, outcome, and study setting) to review the studies. The data extraction form contains the author’s name, publication year, type and study setting, data collection method, sample size, age range, study subject characteristics, outcomes of interest, contextual factors, and findings on the measures of association. The reviewers contacted the authors of the article and requested details through email or the research gate for missing data and an incomplete report. All the searched studies were exported to EndNote X9 software to remove duplication. Afterward, the two authors (MT and WG) independently screened the title and abstract of the studies to identify potentially relevant studies. Next, the retrieved full texts of the study were screened based on the predetermined eligibility criteria and methodology. Finally, the full texts of the selected studies were evaluated for clearly meeting eligibility criteria and reviewed. The details of the screening or selection procedure are stated in Figure 1.

Since the reports of the included studies are minimal and do not report every detail, further analysis was found to be challenging. In order to present the results in a detailed manner, tables and figures were used in addition to narrative reporting of the outcomes.

### Critical appraisal of studies

To evaluate the strength of the recommendations in systematic reviews as well as the quality of the evidence, the GRADE approach was utilized to rate the quality of the eligible studies. Uncertainties were resolved by a joint discussion between the two authors. The GRADE method starts with an explicit query that clearly states all significant and important outcomes. Risk of bias, inconsistency, indirectness of the evidence, imprecision, and publication bias are the key domains utilized to evaluate the certainty of the evidence (15). Accordingly, using the GRADE approach, the majority of the studies that were chosen were not experimental, and their characteristics were rated low (Table 1). The details of the certainty evidence rating were stated in Appendix 2.

**Table 1:** Summary of baseline characteristics of the articles that were published earlier and included in the review, 2023.

Author & publication Year	Study design	Study setting	Average age in years (mean)	Sample size	Data collection tool	Marital status	Gender (Female %)	Occupation	Educational status	Reason for displacement	Grade checklist
Madoro et al. (12)	Cross-sectional	Ethiopia, Gedeo zone	18 to 79 yrs	636	PCL-5	Married – 55.2 Single – 24.3 Divorced – 6.24 Separated – 4.48 Widow/er – 9.76	47.5%	Farmer – 40.3 Merchant – 28.8 Employed (gov’t and private) – 8.8 Student – 18.2 Others – 4.2	No formal education – 22.1 Primary- 47.4 Secondary- 18.56 Preparatory – 9.76 College and above – 2.24	Inter-communal violence along the borders	Low
Salah et al. (7)	Cross sectional	Central Sudan	18–85 years	1,876	Mini International Neuropsychiatric Interview (MINI)	Single-21.9 Married – 69.5 Divorced/widowed-8.6	55.7%	53.4% were unemployed 15.1% with permanent Jobs 47.7% had an income of less than US$100 per month.	Over a fifth (21.6%) had no formal education, 2.3% had a university degree and 53.6% had only up to six years of school education.	War	Low
Hamid et al. (16)	Cross-sectional	Darfur, Sudan	12–85 years	430	PCL-5	74.4% – married 20.5%- single 0.7%- divorced 3.8% – widowed	49.4%	Not reported	Not reported	War	Low
Makango et al. (13)	Cross-sectional	Debre Berhan, Ethiopia	18+ years	406	PCL-5	Single – 17.0 Married – 75.6 Divorced – 6.2 Widowed – 1.2	51.7%	Farmer – 57.4 Civil servant – 6.2 Merchant – 17.5 Student – 5.9 House wife – 9.1 Other – 3.9	Not went to the school – 32.5 Elementary – 41.4 High school – 15.0 Diploma – 6.4 Degree & above – 4.7	Civil war (conflict & war in the northern part of Ethiopia)	Low
Teshome et al. (17)	Cross-sectional	Gondar, Ethiopia	18–72 years	804	PCL-5	Married – 76.4 Single – 16.2 Divorced – 2.5 Windowed – 5.0	46.5%	Gov. employee – 44:3 Daily laborer – 7:0 House wife – 36:1 Merchant – 3:2 Student – 8:2 Others* – 1:2	No formal education – 27.9 Primary school – 41.0 Secondary school – 17.4 Diploma – 9.9 Degree and above – 3.7	Civil war	Low
Rwang et al. (18)	cross sectional study	Jos, Nigeria	18–69 years	248	PTSD-8 Inventory scale	Single – 17.7 Married – 61.3 Widowed – 20.6 Divorced – 0.4	65.3%	Not reported	Adult Education −15.3 Primary School −28.2 Secondary School −40.3 Tertiary Education −11.7 University −13	Conflict between Fulani herdsmen and Berom ethnic group in Jos, north central Nigeria	Low
Sheikh et al. (11)	Cross-sectional study	Kaduna, Nigeria	18–95 years	258	Harvard trauma questionnaire (HTQ)	Married – 59.7 Widowed – 20.5 Single – 13.6 Divorced – 4.3 Separate – 1.9	51.9%	Unemployed – 68.2 Employed = 25.2 Student – 0.8 Retired – 1.2	Quranic – 39.3 Primary – 0.26.5 Secondary – 19.5/ None – 12.1 Tertiary – 2.7	Conflict following the April 2011 elections in Nigeria	Low
Deborah et al. (10)	Cross-sectional	Maiduguri, Nigeria	18+ years	1,200	IES-6	Single – 16.4 Married – 67.4 Divorced – 5.8 Widowed – 10.3	44.9%	Unemployed – 83.3 Employed – 10.1 Student – 4.8 Retired – 1.8	Informal – 74.3 Primary – 8.0 Secondary – 11.3 Tertiary – 6.4	Boko Haram terrorism	Low
Maigari et al. (19)	Cross-sectional	North eastern Nigeria	18+ years	292	MINI International Neuropsychiatric Interview (MINI):	Never married – 58.2 Married −32.2 Previously Married −3.4 Widow – 6.2	30.8%	Group I and II – 1.4 Group III- – 3.42 Group IV -12.3 Group V -24.0 Group VI -58.9	No formal education – 6.8 Primary school – 11.6 Secondary – 51. 4 Tertiary – 30.1	Boko Haram terrorism	Low
Mustafa et al. (14)	Cross-sectional	Somalia	18+ years	401	Harvard Trauma Questionnaire (HTQ)	Single – 5.5 Married – 69.1 Separated – 2 Divorced – 12.5 Widowed – 11.0	83.7%	Employed – 32.2 Unemployed – 66.8 Student – 1.0	None – 64.8 Quranic – 24.2 Primary – 8.5 Secondary – 2.0 Tertiary – 0.5	Armed conflicts and environm ental disasters	Low
Robert et al. (20)	Cross sectional study	Uganda	18+ years.	1,210	Harvard Trauma Questionnaire (HTQ)	married/co-habiting −76.5% single – 6.0% separated −17.5%	60%	Not reported	never attended school −31.4% completed primary school −58.8% completed secondary school −9.8%	Civil Wars	Low

NB: Maigari et al. (19) occupational status: Group I consists of professionals with university degrees (doctors, teachers, lawyers, scientists and high government officers), Group II consists of professionals without university degrees (administrators, high clerical and supervisory personnel’s, large scale farmers, entrepreneurs and armed forces officers), Group III consists of clerks, motor vehicle drivers, mechanics, tailors, butchers, soldiers, police and small-scale entrepreneurs, Group IV consists of barbers, goldsmiths, palm wine tapers and small- scale farmers, Group V includes laborers and petty traders, and Group VI consists of full time house wives, unemployed educated youths and apprentices. This system of classification has been previously used among Nigerians subject.

## Result

In the current review, authors used Embase, PubMed, PsychInfo, and the Web of Science database as search engines to identify relevant studies. Moreover, other sources have also been searched for gray literature. Accordingly, 33,138 articles were initially found in six databases and from other sources. After checking for duplicates and unrelated themes, 2,730 studies were left for further screening. Then 9,202 studies were removed after checking the titles and abstracts. So 25 studies have become the only candidates for full-text review, and finally, the authors have included 11 studies that meet the methodology or eligibility criteria to undertake a systematic review. The majority of studies that met the inclusion criteria in our study made use of validated mental health questionnaires that were collected by trained professionals after assessing their validity and translation into the local language of the study respondents. Accordingly, the DSM-5 PTSD Checklist (PCL-5) was used to measure PTSD in about four studies. In other ways, the Harvard Trauma Questionnaire (HTQ) and the Mini International Neuropsychiatric Interview (MINI) were used in three studies to measure outcome variables.

### Characteristic of studies meet inclusion criteria

A total of 11,010 participants were from fourteen sub-Saharan African countries. Considering the contribution of studies by countries, of the total of 14 studies included in the current review, Nigeria holds the top spot with four studies, followed by Ethiopia and Sudan, which each contributed three studies. Somalia, the Central African Republic, Uganda, and Kenya each contributed single studies. The authors have reviewed studies from February 1, 2023, and June 30, 2023, and almost all of the candidate studies were cross-sectional types other than single articles, which were experimental. In most of the studies (nine studies), female participants dominate, while male participants represent only five studies. All of the studies were conducted from 2008 to 2023. In terms of their occupation, five studies reported from Somalia, Central Sudan, and Nigeria (3) show a greater proportion of unemployed participants compared to employed participants, and the majority of the respondents engaged in private work compared to government service or civil work. The age range of the study respondents ranged from 12 to 95 years. In almost all of the included studies (10 out of 11), married participants dominate the study.

### Prevalence of post-traumatic stress disorder

Post-traumatic stress disorder was the outcome variable studied in this review. The prevalence of post-traumatic stress disorder in sub-Saharan African countries was disproportionately presented in this review, ranging from 12.3% in Central Sudan (7) to 85.5% in Nigeria (19). From a total of 11 studies, eight (6) of them reported more than 50% of the magnitude of post-traumatic stress disorder, pointing to a higher magnitude of PTSD in the study population.

### Determinants of PTSD among IDP in sub-Saharan Africa

The study identified numerous factors that contributed to post-traumatic stress disorder among the internally displaced population. Gender was one of the factors shown to have an association with PTSD. Studies in Ethiopia (12) and Uganda (20) show a female gender association with PTSD. In contrast to this, a study report in Jos, Nigeria (18), shows an association between PTSD and male gender. Regarding the mental health status of the study respondents, depression (11–13, 17), and anxiety (16, 17) were reported in the different studies to have a positive association with PTSD among internally displaced people. In other ways, single marital status (18–20) and a low level of educational status (7, 18, 19) contributed to PTSD compared to their counterparts.

The psychological response of the study respondents in terms of experiencing or witnessing traumatic events is commonly thought to have a significant role in the explanation of internally displaced persons or forced migration. Accordingly, witnessing or seeing trauma, the presence of psychological trauma, rape or sexual abuse (11–14, 17, 20), and a higher frequency of displacement (13, 14) were evidenced for their association with post-traumatic stress disorder among the internally displaced population.

Moreover, age between 18 and 27 years (18), age at first displacement between 19 and 35 years (14), presence of somatic symptoms (17), poor social functioning (16), and being a merchant (13), were also shown to have a statistically significant association with PTSD in IDPs in sub-Saharan Africa.

## Discussion

The current review identified the prevalence of PTSD among IDPs in sub-Saharan Africa. The prevalence of PTSD among IDPs varies by region. The lowest prevalence was seen in Central Sudan (12.3%), whereas the highest was in Nigeria (85.5%). Unlike the current finding, previous evidence found a lower prevalence of PTSD, which ranges from 5.1 to 83.4%. The possible explanation for this variation could be the variation in the characteristics of the study population, as the present review included studies conducted in sub-Saharan African regions. According to the current review, in the majority of findings in the region, the prevalence of PTSD was greater than 50%. This finding is higher compared to a finding from Sri Lanka, where only 2.4% of IDPs developed PTSD (5). This difference might be associated with the variation in accessibility and affordability of mental health care.

The present proportion is higher compared to the two studies conducted in Iraq, where the prevalence was 14.5% PTSD (21, 22). The prevalence in the region was also higher than in Syrian and Kurdish IDPs (23, 24). This high prevalence in the region could be related to different socio-cultural variations and the economic status of the population. The variation might also be related to differences in mental health coverage in the regions.

The second aim of this review was to identify the determinants of PTSD among IDPs in the sub-Saharan region. Accordingly, being female was more associated with PTSD compared to being male. This finding is supported by previous studies conducted in Iraq, Denmark, and Syria (21, 23, 25). This is a plausible correlation that relies on the fact that displaced women were more exposed to the antecedents of PTSD, including gender violence, than men (26–28).

An experience of traumatizing events was also identified as a factor affecting the occurrence of PTSD in the sub-Saharan African region. In the current review, it was found that the frequency or number of traumatic incidents was related to post-traumatic stress disorder, suggesting that repeated exposure to trauma may have an impact on the development of PTSD. This was supported by numerous studies conducted in Iraq (22, 29, 30), Syria (31), Bangladesh (32), Germany (33), and other various settings (34). Furthermore, a study report from Afghanistan (35) and a meta-analysis by Steel et al. (36) reported cumulative trauma as the primary predictor of PTSD, evidencing a dose–response relationship, which is corroborated by the findings of the current review. The current assessment of psychological factors indicated that PTSD was significantly predicted by depression and anxiety disorders in IDP across a number of studies. This was supported by the findings of the Ukrainians, Southern Lebanon (37), Mexico, and other settings (38).

Furthermore, the current review found that the level of education has an association with PTSD among the IDPs. Accordingly, the review showed that low levels of education contribute to PTSD compared to their counterparts. This finding is in contrast to a previous study by Matthew Porter and Nick Haslam, which identified that being more educated enhances PTSD (39). In war-affected areas or refugee camps, older age was shown to have an association with PTSD among IDP (40, 41), in contrast to this study, in which earlier age (between 18 and 27 years) evidenced an association with PTSD comparatively.

The finding of the current review is consistent with a study by Roberts et al. (42) that found single marital status to be one of the socio-demographic indicators that was associated with PTSD among IDP. On the other hand, numerous studies have shown that, among this population, married participants were found to be a risk factor for PTSD (16, 40). The discrepancy might be attributable to how the refugee camps are set up in terms of aid coverage, personal safety, and psychological support.

One of the tragic incidents linked to many psychological squeals, such as torture, sexual abuse or assault, rape, the death of close family members, and conflict, is internal displacement or forced migration. Accordingly, our review discovered a relationship between PTSD and traumatic occurrences, including physical assault, being in a war situation, being tortured or beaten, being forced to embrace beliefs or brainwashed, or witnessing the murder of a stranger among these people. This association was in agreement with the findings of the studies in other settings among displaced populations (43–46). The reason might be due to the fact that the impacts of traumatic events have contributed to PTSD (see Table 2).

**Table 2:** Summary of included studies on the prevalence and determinants of PTSD among internally displaced population in sub-Saharan Africa, 2023.

Author	Prevalence of PTSD	Determinants(significantly associated)
Madoro et al. (12)	366 (58.4%)	Female gender Depression Displaced more than once Destruction of personal property Witness the murder of family Cumulative trauma
Salah et al. (7)	230 (12.3%)	Being displaced for short periods of time Generalized anxiety disorder Having fewer years of education
Hamid et al. (16)	232 (54%)	General distress Somatic symptoms Anxiety Social dysfunction Depression
Makango et al. (13)	274 (67.5%)	Being a merchant Witnessing the destruction of property Facing trauma during displacement Frequency of displacement (3 or more) Being distressed Unemployment
Teshome et al. (17)	3,280 (40.8%)	A close family member killed or seriously injured Being female Moderate and high perceived stress Depression symptoms Anxiety disorder symptoms A chronic medical illness Physical assault and being in a war fighting situation
Rwang et al. (18)	212 (85.5%)	Male gender Age (18–27 years old) Being single Adult level of education (below primarily educational level)
Sheikh et al. (11)	109 (42.2%)	Having a CIDI diagnosis of depression witnessing death of a family member
Deborah et al. (10)	936 (78%)	Not reported
Maigari et al. (19)	186 (63%)	Low level of educational status Single status and Low income before displacement
Mustafa et al. (14)	129 (32%)	Being unemployed Frequency of displacement (more than once) Age at first displacement(19–35 years) Number of traumatic events
Robert et al. (20)	657 (54%)	Female gender, being single, experiencing ill health without medical care, experiencing rape or sexual abuse, Experiencing lack of food or water, and experiencing higher rates of trauma exposure (being tortured or beaten, being made to accept ideas/brainwashing, witnessing murder of stranger or strangers)

### Limitation

The current review summarizes the prevalence of PTSD and its associated factors in sub-Saharan Africa, where internal displacements are common. Despite providing important information on the post-traumatic stress disorder of IDPs in the context of Sub-Saharan Africa, this review contains some shortcomings. For instance, all of the included studies were cross-sectional in nature, and this could possibly raise the issue of temporality and selection bias. Furthermore, small numbers of studies were included, and no meta-analysis was done, so variability among studies, the effect of heterogeneity, and publication bias were not evaluated.

## Conclusion

The prevalence of PTSD in the region is higher compared to other regions of the world. The participants’ socio-demographic characteristics, including age, being single, being female, and having a low level of education, were identified as factors contributing to PTSD. Moreover, the review identified that depression, anxiety, and experiencing or witnessing traumatic events were also influencing factors for PTSD among IDPs. The concerned bodies need to reinforce the monitoring and evaluation of the mental health programs of IDPs in the region.

## Author contributions

TK: Writing – review & editing. MJ: Writing – review & editing, Conceptualization, Data curation, Investigation, Methodology, Software, Supervision, Writing – original draft. MW: Writing – review & editing. JE: Writing – review & editing. AM: Writing – review & editing. AE: Writing – review & editing. LW: Writing – review & editing. SS: Writing – review & editing. MM: Writing – review & editing. DN: Writing – review & editing. GD: Writing – review & editing. WG: Writing – original draft, Writing – review & editing.

## Appendix

Appendix 1 Database search results.

Appendix 2 Details of the certainty evidence ratings.
