# Supplementary material for: A post-traumatic stress disorder among internally displaced people in sub-Saharan Africa: a systematic review
Source: Front Psychiatry. 2023 Nov 3;14:1261230. doi: 10.3389/fpsyt.2023.1261230 (PMC10655091; doi:10.3389/fpsyt.2023.1261230)
Supplement: Supplementary file 2 [file Data_Sheet_2.pdf]

Search strategy

((Posttraumatic stress disorder) OR (posttraumatic stress symptoms) OR (post-traumatic stress disorder) OR (posttraumatic stress symptomatology) OR (PTSD) AND (internally displaced persons) OR (internally displaced peoples) OR (forced migration) OR (internally displaced survivors) OR (IDP) AND (Africa) OR (Sub-Saharan Africa) OR (sub-Saharan African countries))
