# Supplementary material for: A post-traumatic stress disorder among internally displaced people in sub-Saharan Africa: a systematic review
Source: Front Psychiatry. 2023 Nov 3;14:1261230. doi: 10.3389/fpsyt.2023.1261230 (PMC10655091; doi:10.3389/fpsyt.2023.1261230)
Supplement: Supplementary file 3 [file Data_Sheet_3.pdf]

Table 1. Search result of various databases for systematic review

**Database**	**Result**
PubMed/MEDLINE	n=27,161
Scopus	n =123
EMBASE	n=2904
Psych Info	n=700
Web of Science	n=1200
