# Supplementary material for: A post-traumatic stress disorder among internally displaced people in sub-Saharan Africa: a systematic review
Source: Front Psychiatry. 2023 Nov 3;14:1261230. doi: 10.3389/fpsyt.2023.1261230 (PMC10655091; doi:10.3389/fpsyt.2023.1261230)
Supplement: Supplementary file 4 [file Data_Sheet_4.pdf]

GRADE contains four categories of evidence, it is classified as very low, low, moderate, and high (Table 1). These are often named as certainty in evidence or quality of evidence.

Due to latent confounding, evidence derived from randomized controlled trials begins at a high quality and observational data-based evidence as a low quality. Generally, the assessment of each retrieved reviews might be increased or decreased based on various reason. Accordingly, we have stated the score of each studies according to standardized predetermined criteria (Table 1) and most of them fall in low grade ratings as stated below in Table 2.

Table 1. GRADE certainty ratings

**Certainty**	**What it means**
Very low	The true effect is probably markedly different from the estimated effect
Low	The true effect might be markedly different from the estimated effect
Moderate	The authors believe that the true effect is probably close to the estimated effect
High	The authors have a lot of confidence that the true effect is similar to the estimated effect

**Author**	**high**	**Moderate**	**Low**	**Very low**
Madoro D et al (2019)[22]			*	
Salah TT et al (2013)[20]			*	
Hamid AA et al (2010)[23]			*	
Makango B et al (2023)[24]			*	
Teshome A et al (2023)[25]			*	
Rwang GT et al (2018)[26]			*	
Sheikh TL et al (2014)[18]			*	
Deborah O et al 2019[27]			*	
Maigari Y et al (2016)[21]			*	
Mustafa A et al (2021)[17]			*	
Robert B et al (2008)[28]			*	

Table 2: Assessment of quality of evidence of retrieved studies using GRADE Approach
